# Training on insertion and retrieval of optional inferior vena cava filters for interventional radiologists with little or just some experience with the combined use of blood vessel and animal models

**DOI:** 10.1186/2193-1801-2-354

**Published:** 2013-07-30

**Authors:** Takuji Yamagami, Terumitsu Hasebe, Rika Yoshimatsu, Tomohiro Matsumoto, Takeshi Hashimoto, Atsushi Komemushi, Seiji Kamei, Makiyo Hagihara, Yozo Sato, Hiroshi Kondo, Masanori Inoue, Atsuhiro Nakatsuka, Makoto Takahashi, Jun Koizumi, Hiroya Saito

**Affiliations:** Department of Diagnostic Radiology, Institute and Graduate School of Biomedical Sciences, Hiroshima University, 1-2-3 Kasumi, Minami-Ku, Hiroshima, 734-8551 Japan; Department of Radiology, Tokai University Hachioji Hospital, Tokai University School of Medicine, 1838 Ichikawa-machi, Hachioji, Tokyo, 192-0032 Japan; Department of Diagnostic Radiology, Tokai University School of Medicine, 143 Shimokasuya, Isehara, Kanagawa, 259-1193 Japan; Department of Radiology, Kansai Medical University, Moriguchi, Osaka, 570-8507 Japan; Department of Radiology, Aichi Medical University, 1-1, Yazakokarimata, Nagakute, Aichi, 480-1195 Japan; Department of Diagnostic Radiology, Aichi Cancer Center, 1-1 Kanokoden, Chikusa-ku, Nagoya, Aichi, 464-8681 Japan; Department of Radiology, Gifu University School of Medicine, 1-1 Yanagido, Gifu, 501-1194 Japan; Department of Radiology, School of Medicine, Keio University, 35 Shinanomachi, Shinjyuku-ku, Tokyo, 160-8582 Japan; Department of Radiology, Mie University Hospital, 2-174, Edobashi, Tsu, Mie, 514-8507 Japan; Terumo Medical Pranex, Terumo Corporation, 2-44-1 Hatagaya, Shibuya-ku, Tokyo, 151-0072 Japan; Department of Radiology, Sapporo Higashi Tokushukai Hospital, 14-3-1, Kita-33-Hgashi, Higashi-ku, Sapporo, Hokkaido, 065-0033 Japan

**Keywords:** Venous intervention, Inferior vena cava filter, Vena cava, Deep vein thrombosis

## Abstract

**Purpose:**

To evaluate the usefulness of a tool that we developed to simulate performance of insertion and retrieval of optional inferior vena cava filters to be additionally used in training of beginners with an animal model.

**Subjects and methods:**

Thirty young doctors who had little or no experience in insertion and/or retrieval of filters were subjects of this study to evaluate the training tool. Eleven trainees practiced both insertion and retrieval of filters first with the animal model then with the blood vessel model while 19 trainees first practiced with the blood vessel model then with the animal model.

**Results:**

All trainees successfully inserted the filter. Two of the 11 trainees who used the animal model before the blood vessel model failed in retrieval, and 2 of the 19 trainees who used the blood vessel model before the animal model failed. In the former group, mean time for filter implantation and withdrawal in the animal model was 75 ± 62 s and 341 ± 238 s, respectively, and in the latter group were 54 ± 16 s and 311 ± 236 s, respectively.

**Conclusion:**

Training with the combination of a blood vessel model and animal model is helpful for beginners to learn to insert and withdraw optional filters.

## Introduction

In the current medical training program in Japan, we have increasing numbers of trainees rotating through radiology departments in university or academic hospitals, including medical students and junior and senior residents. At the same time, it is increasingly difficult for a radiology trainee to learn invasive angiographic procedures because of the limited training period and concerns about patient safety (Yamagami et al. 2009). Thus, creating opportunities for beginners to learn and intensively practice basic interventional procedures has been needed.

For beginners in interventional radiology, the Japan Society of Interventional Radiology has been holding a hands-on seminar annually to teach procedures that are considered necessary for interventional radiologists. This year insertion and withdrawal of optional filters were themes of this seminar. The training program included use of a combination of a blood vessel model, which was originally designed to simulate performance of insertion and withdrawal of filters, and an animal model. The purpose of the present study was to evaluate the educational effect of this program from a technical perspective and the role of training with a blood vessel model in addition to that with an animal model.

## Materials and methods

### Training tool

The blood vessel model for simulating filter insertion and retrieval mainly replicates the inferior vena cava, right and left renal veins, and right and left iliac veins. Three ringed thin protuberances were made in the part corresponding to the inferior vena cava below the renal vein so that the feet of the filters could be fixed easily to the wall of the model.

**Figure 1:**
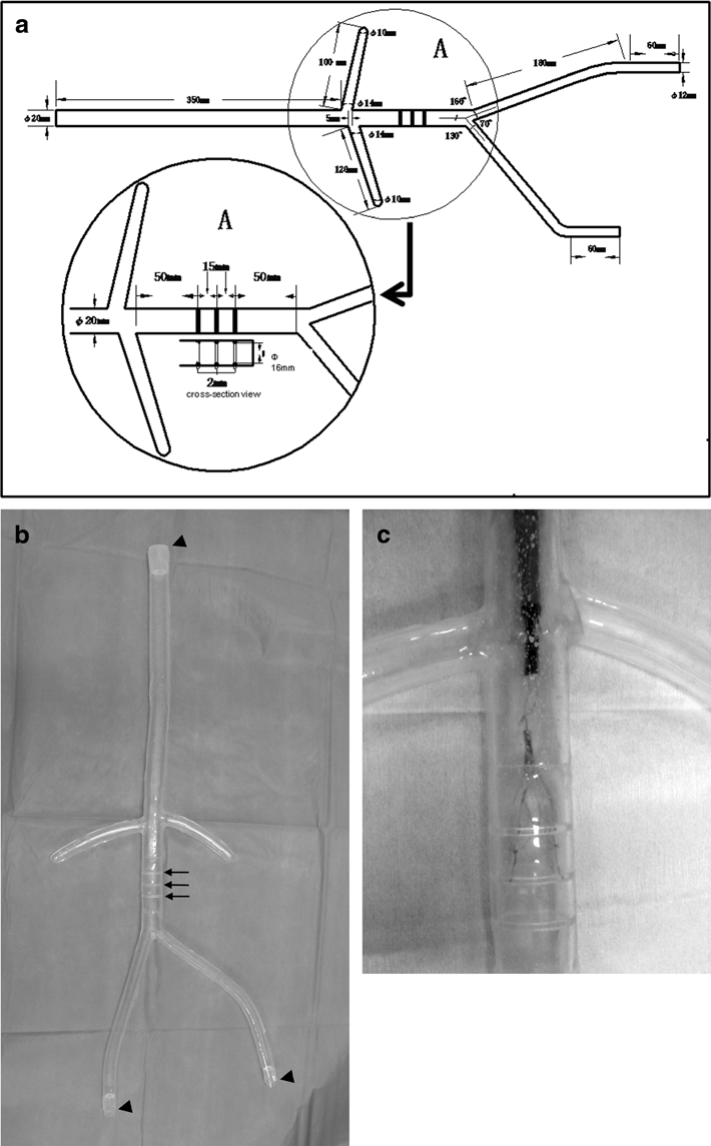
**The blood vessel model. (a)** Design drawing that formed the basis for the training tool. The blood vessel model mainly replicated the central venous system. Note that 3 ringed thin protuberances were made in the part corresponding to the inferior vena cava below the renal vein. **(b)** Photograph shows appearance of the bare model. Holes were created at 3 sites, which are areas corresponding to the cervical and right and left femoral veins, from which a sheath introducer can be inserted (arrows). All holes can be covered when not used. Note the 3 ringed thin protuberances (arrowheads). **(c)** Photograph shows training of withdrawal of the filter with this tool.

This life-size model was made using a three-dimensional computer-aided design system based on a drawing as shown in Figure 1a. The model is made of silicone resin coated internally with silicone oil (Figure 1b). The training tool, which is lightweight and easy to carry, has 3 end holes into which a 14-F sheath introducer can be inserted. Each hole can be covered when not used. Two end holes were used for training. These end holes correspond to entry to the cervical and right femoral veins. Movement of devices such as catheters and guide wires within the vessel is visible from outside of the training tool (Figure 1c). Furthermore, training under the guidance of a computer-controlled display camera that displays the tool and devices is possible.

### Animal model

This study protocol was approved by the institutional Animal Experimental Committee. Three female pigs weighing 50.4–53.0 kg (mean weight 52.1 kg) were studied. All animals were carefully maintained and cared for before and during the experiment in accordance with the guiding principles on care and use of laboratory animals at Terumo Medical Pranex (Kanagawa, Japan). These guiding principles conformed to standards for care and management of experimental animals as established by the Japanese Prime Minister’s office and international guiding principles for biomedical research involving animals (The Japanese association for laboratory animal science (JALAS) 1987).

All procedures were performed with the swine under general anesthesia. Animals were placed in a supine position. Premedication was administered with an intramuscular injection of atropine sulfate (0.05 mg/kg; Tanabe-Mitsubisi-Seiyaku, Osaka, Japan), midazolam (5 mg/kg; Sando Inc., Tokyo, Japan), and xylazine (4 mg/kg; Bayen Health Care, Tokyo, Japan). Anesthesia was induced by thiamylal sodium (5-6.25 mg/kg (iv) Nichikou, Toyama, Japan). After anesthetic administration, an endotracheal tube was inserted, and anesthesia was maintained with sevoflurane (2-4%; Mylane, Osaka, Japan), nitrous oxide (3 l/min), and oxygen (3 l/min). Electrocardiography was used to monitor heart rate and rhythm. Oxygen saturation was monitored using a pulse oxymeter (OGS-2001; Nihon Kohden, Tokyo, Japan). A 7-F vascular sheath was inserted into the femoral artery to monitor real-time blood pressure using a pressure transducer (DTXTM PLUS DT-XX; Nihon Becton Dickinson, Inc., Fukushima, Japan) connected to a pressure polygraph (Cardiomaster RMC 3000; Nihon Kohden). For training with these animal models, a 14-F short sheath was placed into the jugular vein, through which devices for filter implantation and retrieval could be inserted repeatedly. Training procedures were performed using a single-plane fluoroscopy unit (Allura Xper FD20, Philips Electronics Japan, Tokyo, Japan).

### Evaluation of training of beginners with the combination of a training tool and animal model

The effectiveness of training using a combination of a blood vessel model and animal model was evaluated in medical doctors who had little or just some experience as operators in filter insertion and retrieval. A Gunther tulip vena cava filter was used for the training (Cook, Bjaeverskov, Denmark).

Trainees were 30 young interventional radiologists (age: mean 33.4 y, median 33 y, range 28–42 y; years since passing the examination of the National Board of Medical Examiners in Japan, mean 8, median 7, range 3–17) who participated in the 10^th^ academic seminar organized by the Japanese Society of Interventional Radiology at Terumo Medical Pranex held on July 28 and 29, 2012. Mean number of years since first performing interventional radiology was 4.1 (range 0.3 to 14; median 3). As to the number of filter implantations performed among participants as an operator, none had been performed by 11, 1-10 by 17, and more than 10 by 2. Eighteen participants had not performed retrieval while 12 performed 1 to 10 retrievals of an optional filter.

All 5 instructors were experienced in insertion and retrieval of optional filters and were board certified as interventional radiologists by the Japanese Society of Interventional Radiology. Mean number of years of experience as interventional radiologists was 14.2 (range 10 to 21; median 12).

After receiving classroom lectures from instructors on inferior vena cava filters, including those on procedures for implantation and retrieval of filters, trainees practiced with the training tool and animal model. Eleven trainees practiced with the animal model first, then with the blood vessel model while 19 trainees practiced with the blood vessel model first, followed by the animal model. Just after training with the animal model, the 19 trainees were asked to evaluate the usefulness of training with the tool prior to training with the animal model by selecting one of the following possible responses: 1, extremely; 2, quite; 3, moderately; 4, slightly; 5, no.

The trainees practiced using the training tool after one of the five instructors demonstrated implanting and removing the filter using the model. Trainees were allowed to ask advice from their instructor at any time. Instructors also used a hands-on approach as needed in assisting the trainees. This training was done in groups of 9 to 11 trainees. Allotted time for each group was approximately 60 minutes.

In training with the animal model, because the length between the jugular vein and renal vein is longer anatomically compared with the human, it was decided that the filter would be implanted in the inferior vena cava at the cephalad site of entry to the renal vein. After the instructor demonstrated implantation then retrieval of the filter, each trainee took a turn practicing the procedure. The trainees were allowed to ask advice from their instructor at any time. Instructors advised orally; however, they made an effort to let trainees do the procedure by themselves and to assist directly through hands-on instruction as little as possible. The trainee and instructors wore a protective lead apron and radio-protective glasses. An instructor evaluated the procedure on the monitor and timed the procedure. If the trainee could not either implant or retrieve a filter within 13 minutes, the instructor assisted with a hands-on demonstration and allowed the trainee to continue from that point or actually completed the procedure in place of the trainee from that point forward. Time required for implantation and retrieval of the filter, respectively, was measured. When a trainee could not successfully implant or retrieve the filter within 13 minutes, the procedure time was noted as 13 minutes.

## Results

Filters were successfully inserted into the inferior vena cava in the animal model by all 30 trainees with a mean time of 62 ± 40 (SD) s (range 31-230 s). On the other hand, filters were successfully retrieved without hands-on assistance by instructors within the time limit by 26 trainees (87%) at a mean time of 322 ± 233 (SD) s (range 79-780 s).

**Figure 2:**
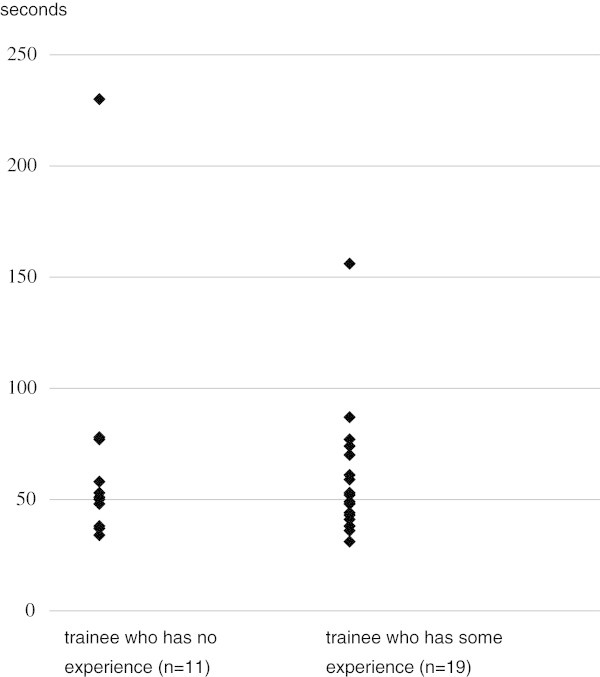
**Comparison of time of filter implantation in the animal model between trainees who had or had not implantation experience.**

**Figure 3:**
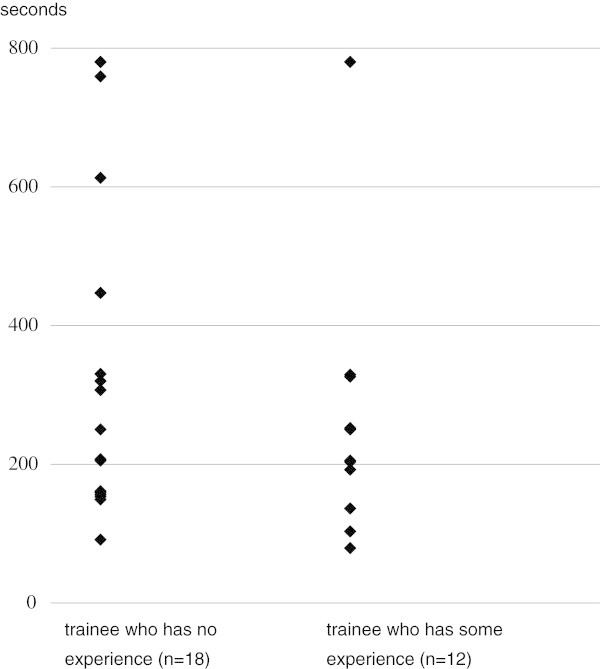
**Comparison of time of retrieval of the filter in the animal model between trainees who had and had not experience in retrieval.**

We compared the time required for filter implantation according to whether or not the trainee had experience in this procedure. Mean time for implantation of the filter in the animal model was 69 ± 55 (SD) s (range 34-230 s) in the 11 trainees without experience in implantation and 58 ± 28 (SD) s (range 31-156 s) in 19 trainees having such experience, with no significant difference between groups (p = 0. 8127 by Mann-Whitney’s U test) (Figure 2). As to retrieval of the filter, 2 (11.1%) of the 18 trainees without experience failed in retrieval in the animal model, while 2 (16.7%) of 12 trainees having some experience failed. The difference between groups was not significant (p = 0.6610 according to chi square test). Mean time for removal of the filter in the animal model was 335 ± 237 (SD) s (range 91-780 s) for the 18 trainees without experience and 303 ± 236 (SD) s (range 79-780 s) for 12 trainees having some experience in retrieval (Figure 3). No significant difference was noted between groups (p = 0.7666 by Mann-Whitney’s U test).

**Figure 4:**
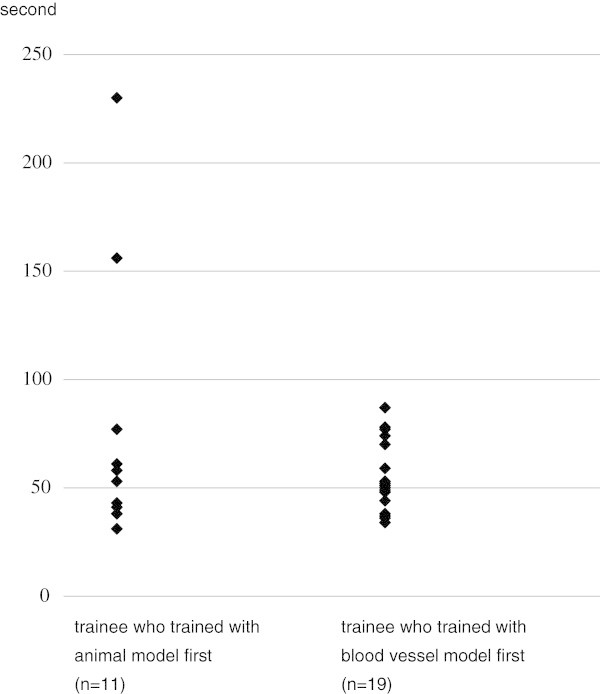
**Comparison of time for implantation of the filter in the animal model between trainees who trained with the animal model first and trainees who trained with the blood vessel model first.**

In the group in which training with the animal model was done before training with the blood vessel model (n = 11), mean time for filter implantation was 75 ± 62 (SD) s (range 31-230 s), while the mean time was 54 ± 16 (SD) s (range 34-87 s) in the group that trained with the blood vessel model before the animal model (n = 19) (Figure 4). There was a tendency for more rapid implantation in the latter group although the difference was not statistically significant (p = 0.8463 by Mann-Whitney’s U test).

**Figure 5:**
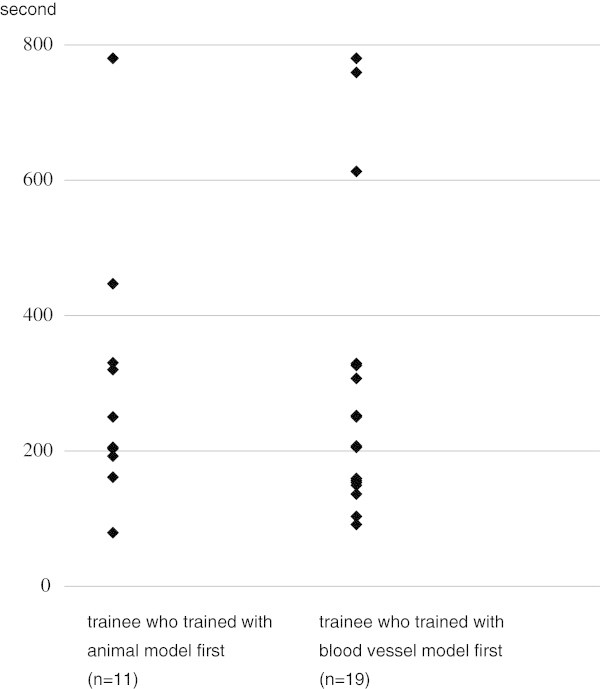
**Comparison of time for retrieval of the filter in the animal model between trainees who trained with the animal model first and trainees who trained with the blood vessel model first.**

Two (18.2%) of the 11 trainees who trained with the animal model prior to that with the blood vessel model failed to retrieve the filter within the time limit while 2 (10.5%) of the 19 trainees who trained first with the blood vessel model also failed, with no significant difference between groups(p = 0.5522 by chi square test). In the former group (n = 11), the mean time for filter retrieval in the animal model was 341 ± 238 (SD) s (range 79-780 s) and was 311 ± 236 (SD) s (range 91-780 s) in the latter group. A tendency for a more rapid retrieval was shown in the latter group, but the difference was not significant (p = 0.4507 by Mann-Whitney’s U test) (Figure 5).

In the 12 trainees with experience in retrieval of a filter, the mean time for retrieval in the animal model was 292 ± 278 s in those who trained with the animal model prior to training with the blood vessel model (n = 5) and 311 ± 224 s in those who first trained with the blood vessel model (n = 7). The remaining 18 trainees had no experience in retrieval. Among them, the mean retrieval time with the animal model was 381 ± 217 s in those who trained with the animal model prior to training with the blood vessel model (n = 6), while it was 312 ± 253 s in those who first trained with the blood vessel model (n = 12).

Opinions as to the helpfulness of using the blood vessel model prior to training with the animal model given by the 19 trainees who initially trained with the blood vessel model were as follows: extremely, n = 17; quite, n = 2; moderately, n = 0; slightly, n = 0; no, n = 0.

## Discussion

There are 3 types of filters: permanent, temporary, or optional. When only short-term protection is required, ideally a permanent inferior vena cava filter would not be placed, particularly if a long life is expected for the patient, considering the demerits of permanent filter implantation with high frequency of recurrence of DVT (Decousus et al. 1998). Thus, a temporary vena cava filter has been widely used for this purpose (Neuerburg & Günther 1994; Lorch et al. 2000). However, paralleling the increased use of temporary vena cava filters, complications have been described that were mainly associated with their structure, in that part of the device projects from the insertion site (Lorch et al. 2000; Carcone et al. 1995; Stosslein & Altmann 1998). Some of these complications were serious, and included infection where the device protruded from the insertion site (Millward et al. 2001), air embolism through a defective sheath (Lorch et al. 2000), worsening of proximal thrombosis along the attached catheter (Carcone et al. 1995), and migration of the filter into the pulmonary artery (Stosslein & Altmann 1998). Moreover, temporary filters must be replaced by permanent filters (Lorch et al. 2000) when the maximal implantation period is reached before successful completion of therapy for DVT. Because of the above-mentioned complications and problems with temporary vena cava filters, the use of an optional vena cava filter that could be implanted without an attached catheter or guide wire would be advantageous. If necessary, this filter could also serve as a permanent filter (Yamagami et al. 2006; Yamagami et al. 2008). Although previous reports described a broad range of rates of removal of optional filters from 1.0 to 40.5% (Yamagami et al. Yunus et al. 2008; Janjua et al. 2010; Helling et al. 2009; Johnson et al. 2010; Gaspard & Gaspard 2009; Zakhary et al. 2008; Kalva et al. 2006; Rimon et al. 2011; Charles et al. 2009), removal rates have been rapidly increasing. For example, Minocha et al (Minocha et al. 2010) reported a recent rate of filter retrieval reaching 60%. Hence, skills not only for implantation of optional filters but also for their withdrawal are required for interventional radiologists.

Traditionally, endovascular skills are learned under direct supervision and guidance with human patients in the catheterization laboratory. This setting, commonly referred to as the master/apprentice model, remains the most common training ground to develop a safe set of skills for use in interventional radiology (Berry et al. 2008). However, training beginners in an invasive procedure in a clinical setting is difficult from the perspective of patient safety.

To address this problem, non-clinical training grounds have been reported. Those reported are roughly classified into two types: animal laboratories and virtual reality laboratories (Berry et al. 2008). The former provides training with the use of anesthetized animals, into which interventional equipment is inserted (Berry et al. 2008). The latter offers ex vivo training with modified interventional equipment with which trainees treat computer-simulated patients (Berry et al. 2008; Patal & Gould 2006; Wang et al. 2007). However, both have some unavoidable demerits, such as cost, inconvenience, and difficulty in portability.

In the present study, we provided training on implantation and retrieval of vena cava filters with animal models for interventional radiologists with little or just some experience in filter implantation and retrieval in only 2 days. Because of the limitations of the two-day training period as well as the large number of trainees, to increase the efficiency of our training effort we used a blood vessel training model in addition to an animal model. This novel tool that we developed is inexpensive compared to the use of animal laboratories and virtual reality laboratories, is convenient to use, and is portable. That our training tool improved the quality of training using an animal model was demonstrated by the fact that the mean time for both inserting and withdrawing optional filters was shorter in the group in which training with our blood vessel model was done prior to use of the animal model than in the group that first used the animal model. It is noteworthy that even in trainees without experience in withdrawal of an optional filter; the mean time of withdrawal in the animal model was relatively short, especially in the group that first trained with the blood vessel model. Additionally, training with this blood vessel model retains the master/apprentice concept. In fact, response of trainees to the question regarding the usefulness of this tool for training combined with use of the animal model was uniformly positive.

We would like to emphasize that daily training with human patients under the master/apprentice model is required for a physician to become an experienced interventional radiologist. However, as initial training, that with an animal model is recommended as one of the more ideal training methods. At the time of use of an animal model, additional usage of a blood vessel model, even a very simple and non-computerized one, would increase the quality of training.
